# Efficacy of autologous stem cell therapy in femoral head avascular necrosis: a comparative study

**DOI:** 10.1186/s13018-023-04297-0

**Published:** 2023-10-24

**Authors:** İbrahim Ulusoy, Mehmet Yılmaz, Aybars Kıvrak

**Affiliations:** 1 Selahhadin Eyyubi State Hospital, Diyarbakır, Turkey; 2 Deva Hospital, Gaziantep, Turkey; 3 Adana Avrupa Hospital, Adana, Turkey

**Keywords:** Avascular necrosis, Femoral head, Stem cell implantation, HHS, VAS, Total hip arthroplasty

## Abstract

**Objective:**

Avascular necrosis of the femoral head is a disease usually seen in middle-aged individuals. Although many aetiological factors have been blamed, there are still aetiological factors that have not been fully elucidated. Although treatment options show a wide range, early and appropriate treatment is of great importance to preserve the hip joint. In our study, we compared the results of core decompression and core decompression combined with bone marrow mesenchymal stem cell implantation in patients with avascular necrosis of the femoral head.

**Material method:**

In this retrospective study, Steinberg stage 1–2 patients operated on for avascular necrosis of the femoral head between 2018 and 2023 were analysed. Separate groups were formed from patients who underwent isolated core decompression and core decompression + bone marrow mesenchymal stem cell implantation. Age, gender, Steinberg staging, aetiology of the disease, follow-up period, progression to hip arthroplasty, Vas scores, Harris hip scores (HHS), and complications were evaluated. Harris hip scores at preoperative and 2-year follow-up periods; VAS scores at preoperative, 3-month, 6-month, 1-year, and 2-year follow-up periods were analysed.

**Results:**

In the study, 44 patients were analysed. While 25 patients underwent core decompression only (group 1), 19 patients underwent core decompression and bone marrow mesenchymal stem cell implantation (group 2). The mean age of the patients in group 1 was 39.3 ± 6.5 years, and the mean age of the patients in group 2 was 38.4 ± 6.7 years. The mean follow-up was 31.85 ± 4.4 months in group 1 and 32.2 ± 4.1 months in group 2. Total hip arthroplasty was performed in 2 of the patients in group 1 (one of the patients underwent total hip arthroplasty at month 28 and the other at month 33).

**Conclusion:**

The treatment of avascular necrosis of the femoral head varies according to various staging methods. Early diagnosis of the disease and correct treatment are very important for the patient's quality of life in the future. In our research, we found that patients who received both core decompression and stem cell implantation for early-stage avascular necrosis of the femoral head exhibited decreased pain at the 6-month, 1-year, and 2-year follow-up examinations. Additionally, their hip function improved at the 24-month mark according to the HHS evaluation.

## Introduction

Avascular necrosis (AVN) of the femoral head is a pathology that occurs as a result of decreased blood flow in the bone due to many different aetiological causes such as steroid use, alcoholism, and trauma [1]. Generally, the diagnosis is made with MRI, which has high sensitivity and specificity in patients presenting with hip pain.

Many classification systems have been defined for follow-up and treatment. The most commonly used classification methods are Steinberg, Ficat–Arlet, Association Research Classification Osseous (ARCO), and Ohzono classification [2]. The Fıcat–Arlet classification, which is most commonly used in practice, is based on scintigraphy, and in this classification, lesion size and joint involvement are not taken into account. With the introduction of magnetic resonance imaging (MRI) and the use of early-stage treatment models, the deficiencies in the Ficat–Arlet classification are being tried to be eliminated with the Steinberg classification consisting of 7 stages using MRI [3].

Since the risk of chondral fractures, collapse, and osteoarthritis is high in untreated cases, early diagnosis and correct treatment are of great importance.

In its treatment, surgical and non-surgical treatments are preferred depending on the aetiological reasons, stage of the disease, duration of symptoms, pain, age, general condition, and whether the disease is unilateral or bilateral. Treatment consists of bisphosphonate therapy, hyperbaric oxygen, electrical stimulation, extracorporeal shock wave therapy, bone marrow mononuclear cell implantation, core decompression, and total hip arthroplasty [4].

Core decompression is one of the most commonly used methods in surgical treatments [5].

Core decompression is usually performed by puncturing and removing a segment of the necrotic lesion. Technical modifications such as multiple drilling, combining with bone grafting, or administering growth factors and bone marrow cells have been proposed [6].

Many studies are showing that core decompression alone has a low chance of success [7]. Especially in young patients, the results of its application alone have not been found to be superior to non-surgical treatment methods [8].

In addition, publications are showing that osteoblast proliferation capacity decreases, bone cell apoptosis increases, and mesenchymal stem cell activity in the bone marrow decreases in cases of avascular necrosis of the femoral head [7].

In our research, we compared the functional outcomes of patients with early-stage femoral head avascular necrosis who underwent simple core decompression and core decompression + bone marrow mesenchymal stem cell implantation.

## Material method

The study was carried out with the permission of the clinical ethics committee. This retrospective study analysed the medical records of patients who underwent bone marrow mesenchymal stem cell implantation with core decompression or core decompression alone for avascular necrosis of the femoral head between 2018 and 2023.

Steinberg classification was used in our study. MR images and radiographs were used in the diagnosis and Steinberg staging of the patients. Criteria for inclusion in the study were defined as age between 18 and 65, Steinberg stage 1–2, and follow-up duration of more than 2 years. [9].

Patients with a history of previous hip surgery, and suspicious or incomplete information in the hospital information system were not included in the study. Age, gender, Steinberg staging, aetiology of the disease, follow-up period, progression to hip arthroplasty, Vas scores, Harris hip scores (HHS), and complications were evaluated.

Surgical technique.

Core decompression was performed under fluoroscopy in all patients. Before surgery, the location of the lesion was determined by measuring the patients' MRI and radiographs. The images measured during the surgery were projected on the screen. The area corresponding to the area where the measurement was made on the scope was targeted, and the lesion area was reached by entering from the lateral femur with a 2-mm k-wire. A single channel was opened over the K wire with a 10-mm cannulated drill. Necrotic tissue, whose borders were previously measured, was removed from the opened channel with the help of a moving-head curette. It was confirmed that all areas measured with scopy were reached.

The prepared bone marrow concentrate was injected into the opened canal, and the exit point of the canal was closed with the help of autologous bone graft. Patients were allowed to walk without weight-bearing at 3 weeks post-operatively. Partial weight bearing was started at 3–6 weeks, and full weight bearing was started after 6 weeks.

Preparation of bone marrow concentrate.

The adjacent iliac crest area is sterile-draped. Using the Arthrex Angel BMAC system (Arthrex, Inc., Naples, FL, USA), two 30-ml syringes are filled with bone marrow aspirate pre-treated with 5 ml acid citrate dextrose using the appropriate cannula. The sample is then processed using the Angel system according to the manufacturer's instructions. After centrifugation for approximately 17 min, the concentrated content is mixed with 5 ml of demineralised bone matrix to obtain a pasty concentrate.

Outcome measures.

**Fig. 1:**
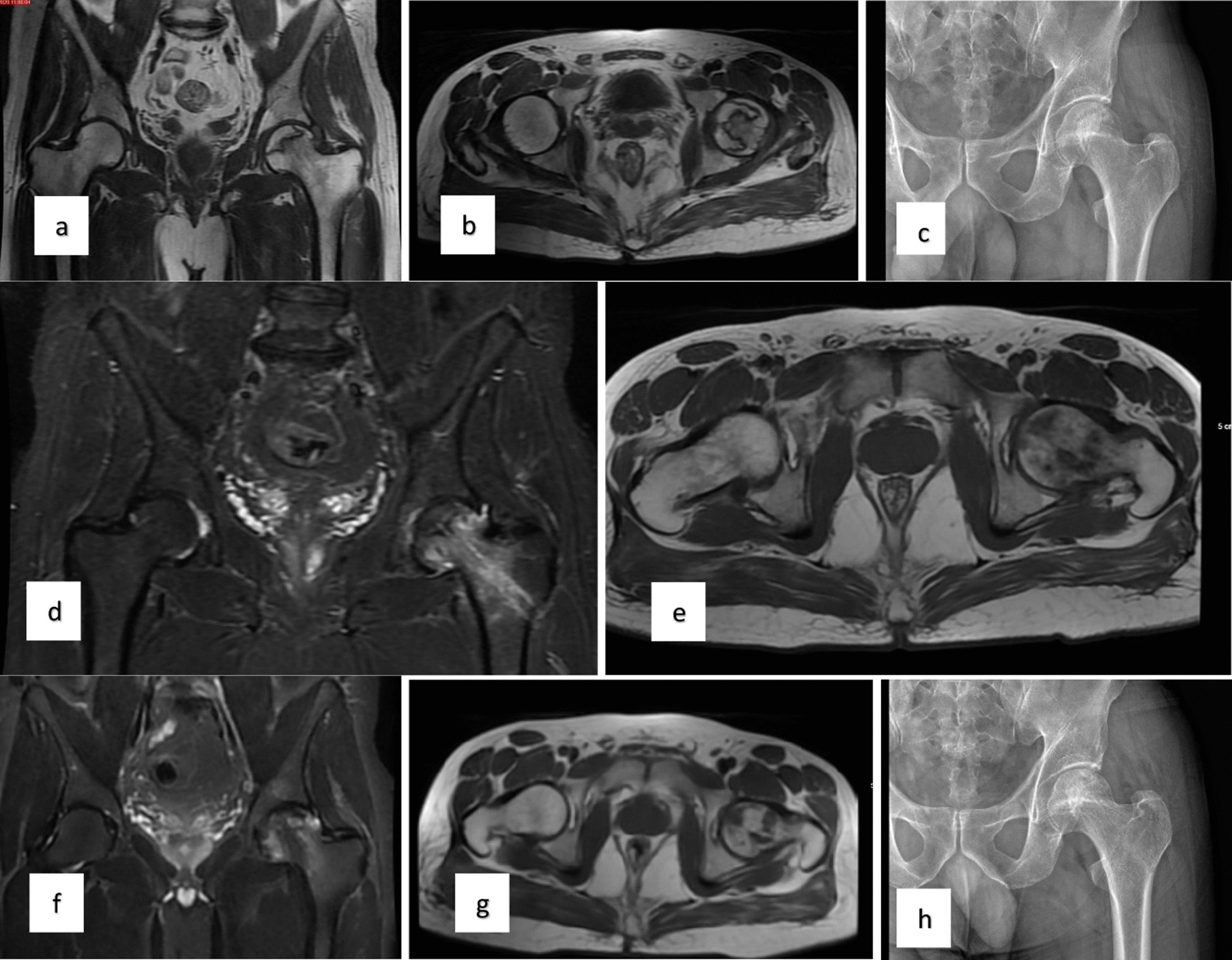
**a**–**c** Preoperative MR and X-ray images of the patient with left femoral head avascular necrosis. **d**, **e** MR images at 3 months after surgery** f**–**h**: MR and X-ray images at second year after surgery

Preoperative, 3-month, 6-month, 1-year, and 2-year follow-ups of the patients were evaluated (Fig. 1). VAS scores were routinely checked during the follow-up. VAS score is scoring that scores the patient's pain between 0 and 10. Harris hip scores (HHSs) were also evaluated in the follow-up of the patients. In this scoring, a score between 0 and 100 is made according to the answers given to the questions. A high score indicates good results, and a low score indicates poor results. In addition, the hips of the patients were evaluated by direct radiography during follow-up. Statistical analyses were performed using the Statistical Package of the Social Sciences (IBM SPSS 28.0.1.0; Corp., Armonk, NY, USA). The variables were investigated using visual (histograms, probability plots) and analytical methods (Kolmogorov–Smirnov/Shapiro–Wilk's test) to determine whether or not they are normally distributed. Descriptive analyses were presented using means and standard deviations for normally distributed variables. The Student's t-test was used to compare the parameters. The Chi-square test or Fisher’s exact test was used to compare proportions in different groups. These tests were chosen within the framework of the general rules in statistics, depending on the characteristics of the dependent and independent variables A *p*-value of less than 0.05 was considered to show a statistically significant result.

## Results

As a result of the search, data were obtained on 57 patients. A total of 44 patients were included in the study after considering the exclusion criteria. While 25 patients underwent core decompression only (group 1), 19 patients underwent core decompression and bone marrow mesenchymal stem cell implantation (group 2).

In group 1, 11 patients had Steinberg stage 1 and 14 patients had stage 2; in group 2, 10 patients had stage 1 and 9 patients had stage 2 AVN of the femoral head. There was no significant statistical difference between the two groups in terms of staging (*p* = 0.57).

The mean age of the patients in group 1 was 39.3 ± 6.5 years, and the mean age of the patients in group 2 was 38.4 ± 6.7 years. The age of the patients in the two groups was statistically similar (*p* = 0.689).

Risk factors in Group 1; The cause was alcohol in 2 patients, steroids in 4 patients, idiopathic in 16 patients, sickle cell anaemia in 1 patient, and post-traumatic femoral neck fracture in 2 patients. In Group 2, risk factors were alcohol in 3 patients, steroids in 10 patients, idiopathic in 5 patients, and post-traumatic femoral neck fracture in 1 patient.

The mean follow-up was 31.85 ± 4.4 months in group 1 and 32.2 ± 4.1 months in group 2. The follow-up time was statistically similar between the groups (*p* = 0.749). Total hip arthroplasty was performed in 2 of the patients in group 1. The patient ages were 44 and 49. The initial stage of the patients is Steinberg 2. The gender of the patients was male, and they did not have osteoarthritis before. There is trauma to the aetiology of both patients. (One of the patients underwent total hip arthroplasty at month 28 and the other at month 33.)

**Fig. 2:**
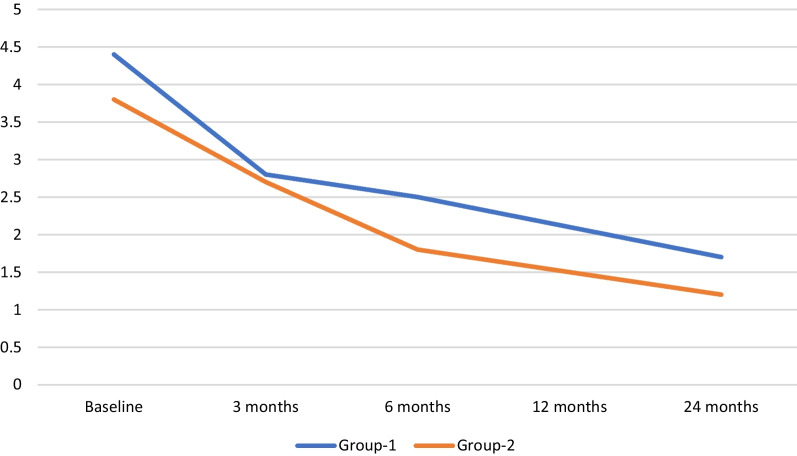
Change in patients' VAS scores

When the VAS scores of group 1 and group 2 were evaluated, it was concluded that the VAS scores were similar at baseline and month 3; there was a statistically significant difference at 6, 12, and 24 months, and the VAS scores of group 2 were significantly lower. When comparing the HHS of the patients in the groups, it was concluded that the baseline scores were similar and there was a significant improvement in group 2 at month 24. In addition, HHS improved significantly before and after treatment in both groups. The changes in VAS scores of patients in group 1 and group 2 are shown in Table 1, and the HHS scores are shown in Table 2 (Fig. 2).

**Table 1:** Change in patients' VAS scores

	VAS-baseline	VAS-3 months	VAS- 6 months	VAS-12 months	VAS-24 months
Group 1	4.4 ± 1.3	2.8 ± 0.8	2.5 ± 0.6	2.1 ± 0.6	1.7 ± 0.5
Group 2	3.8 ± 1.1	2.7 ± 0.7	1.8 ± 0.4	1.5 ± 0.6	1.2 ± 0.6
*p* value	0.215	0.556	< 0.001	0.002	0.003

*VAS* Visual Analog score

**Table 2:** Post-treatment change in patients' HHS

	HHS-baseline	HHS-24 months	*p* value^1^
Group 1	68.36 ± 6.7	81.15 ± 2.2	< 0.001
Group 2	67.76 ± 5.7	83.69 ± 4.9	< 0.001
*p* value^2^	0.757	0.049	

^1^Statistical comparison of values in the same group of patients before and after treatment

^2^Statistical comparison of baseline and 24-month HHS between the two patient groups

*HHS* Harris Hip Score

## Discussion

The treatment of AVN of the femoral head varies according to various staging methods. Early diagnosis of the disease and correct treatment are very important for the patient's quality of life in the future.

In our study, HHS was observed to be good in both patient groups. In all postoperative follow-ups, it was observed that the patients had no movement restrictions. Pain was at the forefront in the patients.

We found that patients who received both core decompression and stem cell implantation for early-stage avascular necrosis of the femoral head exhibited decreased pain at the 6-month, 1-year, and 2-year follow-up examinations. Additionally, their hip function improved at the 24-month mark according to the HHS evaluation.

Core decompression is a surgical technique that aims to relieve oedema and improve circulation by reducing pressure on the femoral head. Its clinical results alone are still controversial in the current literature due to its success rate, especially in collapse phase cases [10]. In recent years, there has been excitement about the application of osteogenic precursors to necrotic lesions [11]. A 25-year study conducted by Hernigou showed that stem cell therapy reduced collapse and THA conversion rate compared with core decompression alone [12]. In their study, Li M and colleagues supported the use of stem cells because they provided better 10-year subjective assessment scores and longer median survival time [13].

In AVN of the femoral head, the disease usually leads to bone collapse and the development of osteoarthritis [1]. Therefore, early diagnosis and treatment of this pathology are important. Many factors determine the treatment of AVN of the femoral head. The stage of the disease, reversible or irreversible aetiological factors are some of them. In the early stages, core decompression is a common surgical treatment in the absence of femoral head collapse. During core decompression, many different treatment methods have been described in the literature. Traditionally, core decompression is performed using a trephine to open an 8–10-mm-wide canal [14]. However, percutaneous methods such as multiple small drills have also been described in the literatüre [6, 15, 16]. In addition, there are articles recommending core decompression with electrical stimulation [17]. There are also articles on the use of vascularised or non-vascularised bone grafts with core decompression [18]. Although there are authors who advocate the superiority of multiple drills, there is no clear evidence in the literature that these methods are superior [6, 16, 19, 20]. Therefore, in light of the literature, we can say that the core decompression method performed by opening an 8–10-mm-wide canal, which is traditionally used in our patients, is an appropriate application.

Despite core decompression being one of the most commonly used treatments in the early stages, its limited effectiveness has led clinicians to look for an alternative treatment. Combined with stem cell application, stem cell therapy appears to be an alternative treatment method. Stem cells can be derived from many tissues. Autologous bone marrow, adipose tissue and dental pulp are some of them. Many studies on stem cells have found that they induce new bone formation and neovascularisation [1]. We believe that this is the mechanism behind the greater reduction in pain and improved HHS in patients who have undergone core decompression and stem cell implantation.

Core decompression + bone marrow implantation has some advantages and disadvantages. The main advantages are that it is minimally invasive, aims to preserve the hip joint, has a wide range of revision options in the event of failure and is combined with a biological solution. On the other hand, the fact that the bone marrow aspirate has to go through certain procedures during the operation, which is time-consuming, and that the bone marrow aspirate area creates an additional incision site, can be counted among the disadvantages.

Looking at the literature, most studies evaluated patients undergoing total hip replacement after AVN [21–23].

When we look at the data about the patients, there is no relationship between age, gender, aetiology and location of the lesion and going for total hip replacement. In our study, it was observed that two patients who required total hip had trauma in their aetiology.

However, only operated patients were included in these evaluations. Patients who were indicated for total hip replacement but could not be operated on for any reason were not included. Patients may avoid total hip replacement due to fear of surgery, religious beliefs, fear of implantation, or other socio-cultural factors [24, 25]. In our study group, apart from two patients who underwent total hip arthroplasty, there were no other patients with an indication for total hip arthroplasty.

One of the major limitations of our study is its retrospective nature. In addition, the aetiology of avascular necrosis of the femoral head is at the forefront of treatment planning. Large groups of patients with the same aetiology will minimise the number of variables. Another limitation is that follow-up was limited to 2 years. Longer follow-up will increase the value of the study.

## Conclusion

If avascular necrosis of the femoral head is not treated early and appropriately, the development of hip osteoarthritis and subsequent total hip arthroplasty may be an inevitable outcome. Especially in early-stage patients, core decompression and the application of combined biological solutions will help protect bone tissue along with physiological remodelling and stop the progression of the disease.

## Data Availability

The datasets used and analysed during the current study are available from the corresponding author on reasonable request.
